# Phytochemical Profile and Anticancer Potential of Endophytic Microorganisms from Liverwort Species, *Marchantia polymorpha* L.

**DOI:** 10.3390/molecules27010153

**Published:** 2021-12-28

**Authors:** Mateusz Stelmasiewicz, Łukasz Świątek, Agnieszka Ludwiczuk

**Affiliations:** 1 Department of Pharmacognosy with the Medicinal Plant Garden, Medical University of Lublin, 20-093 Lublin, Poland; aludwiczuk@pharmacognosy.org; 2 Department of Virology with SARS Laboratory, Medical University of Lublin, 20-093 Lublin, Poland; lukasz.swiatek@umlub.pl

**Keywords:** liverworts, *Marchantia polymorpha*, endophytes, diketopiperazine, sesquiterpenoids, anticancer activity

## Abstract

Liverwort endophytes could be a source of new biologically active substances, especially when these spore-forming plants are known to produce compounds that are not found in other living organisms. Despite the significant development of plant endophytes research, there are only a few studies describing liverwort endophytic microorganisms and their metabolites. In the presented study, the analysis of the volatile compounds obtained from thallose liverwort species, *Marchantia polymorpha* L., and its endophytes was carried out. For this purpose, non-polar extracts of plant material and symbiotic microorganisms were obtained. The extracts were analyzed using gas chromatography coupled to mass spectrometry. Compounds with the structure of diketopiperazine in the endophyte extract were identified. Liverwort volatile extract was a rich source of cuparane-, chamigrane-, acorane-, and thujopsane-type sesquiterpenoids. The cytotoxicity of ethyl acetate extracts from endophytic microorganisms was evaluated on a panel of cancer (FaDu, HeLa, and SCC-25) cell lines and normal (VERO), and revealed significant anticancer potential towards hypopharyngeal squamous cell carcinoma and cervical adenocarcinoma.

## 1. Introduction

Endophytes are microorganisms (bacteria or fungi) that spend all or part of their life colonizing cells and the intercellular spaces of plant tissues, without causing apparent harm to the plant. The effects of these organisms on the host may include combating pathogenic microorganisms, inducing immunity, promoting plant growth and development by binding free nitrogen, synthesizing phytostimulants, increasing the uptake of minerals, and increasing plant resistance to adverse abiotic factors [1,2,3]. Therefore, these microorganisms are also a potential source of metabolites showing various biological properties. Some endophytes are capable of producing own substances with antibacterial, antifungal, antiviral or cytostatic properties. In particular cases, endophytes may produce compounds similar or the same as the host plant [2,4,5]. An example is taxol, a highly cytotoxic plant metabolite, and the first anticancer agent obtained from endophytic fungi, *Taxomyces andreanae* colonizing *Taxus brevifoilia*. Another bacterial endophyte is *Streptomyces* NRRL 30562, isolated from *Kennedia nigrican* cells. Produced by this bacteria, munumbicin is a compound that shows a strong antibacterial activity against gram-positive strains of *Bacillus anthracis*, resistant strains of *Mycobacterium tuberculosis*, and also inhibits the development cycle of *Plasmodium falciparum*, a unicellular protozoan parasite responsible for malaria [6].

Almost all vascular plants are colonized by bacteria or fungi. Marchantiophyta (liverworts), Bryophyta (mosses), and Anthocerotophyta (hornworts) also live in symbiosis with endophytes. The bryophytes remain a poorly studied area in terms of secondary metabolites in comparison with vascular plants. This is a huge niche that can be a source of new biologically active compounds [7]. Among bryophytes, the liverworts are a group of about 6000 species of spore-forming plants which can be found all over the world, growing on wet soil, rocks, tree trunks, lakes, and rivers. According to scientific data, liverworts are the ancestors of all known land plants. They appeared 472 million years ago [8]. A characteristic feature of nearly all liverworts is that they have oil bodies, which are intracellular organelles surrounded by a one-piece membrane. These structures are filled with volatile aromatic compounds, mainly terpenoids [9,10,11].

Although the beginning of research on endophytes dates back to the 19th century, there are still many plant species not studied in this regard. Among them is *Marchantia polymorpha* L., one of the most popular liverwort species in the world. Many studies have shown beneficial properties of this liverwort, including antipyretic, antibacterial, diuretic, and hepatoprotective properties. Additionally, *M. polymorpha* is successfully used in the treatment of cuts, fractures, burns, snake bites, and various types of open wounds [10]. A detailed phytochemical analysis of this species has also been carried out, and compounds belonging to group of cyclic bis-bibenzyls, sesquiterpenoids, and diterpenoids have been identified and/or isolated [11]. However, none of the previous studies focused on isolation and evaluation of the properties of compounds produced by endophytic microorganisms of this liverwort species. Therefore, the aim of the present study was to perform a phytochemical analysis of metabolites present in endophytes of the *Marchantia polymorpha* L. Moreover, the cytotoxicity and anticancer potential of ethyl acetate extract from endophytic microorganisms isolated from *M. polymorpha* was evaluated. 

## 2. Results

### 2.1. GC-MS Analysis of the Volatiles Present in Thalli of Marchantia polymorpha

**Figure 1:**
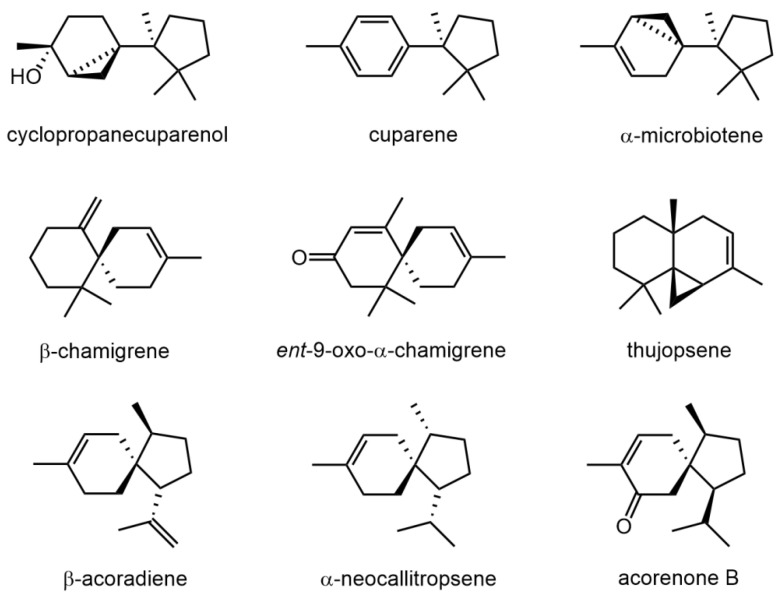
Structures of the most characteristic volatile components found in thalli of *Marchantia polymorpha*.

Gas chromatography coupled to mass spectrometry of diethyl ether extract obtained from the thalli of *Marchantia polymorpha* allowed for identification of characteristic volatile components. The data presented in Table 1 confirmed the presence of sesquiterpenes as the major compounds of the non-polar extract. The most characteristic volatile metabolites are two sesquiterpene alcohols belonging to cuparane type: cyclopropanecuparenol and its diastereoisomer, *epi*-cyclopropanecuparenol. The relative percentage of these components, among all detected, was 10.8% and 22.3%, respectively. Besides the mentioned alcohols, other cuparane-type sesquiterpenes have also been identified. These are cuparene, δ-cuprenene, cuparophenol, and α- and β-microbiotene. The second group of sesquiterpenoids detected in this liverwort species were chamigrane-type compounds, represented by β-chamigrene and ent-9-oxo-α-chamigrene. Besides cuparanes and chamigranes, the presence of acorane-type sesquiterpenoids, e.g., α-neocallitropsene, acorenone B, β-alaskene, and β-acoradiene, were confirmed. The identified diterpenes included: neophytadiene and phytol. These compounds are typically found in all green plants, as they are products of chlorophyll degradation. The structures of the most characteristic volatile compounds found in the thalli of *M. polymorpha* are presented on Figure 1. 

### 2.2. GC-MS Analysis of the Volatile Components Present in Endophytic Microorganisms from Thalli of M. polymorpha 

**Figure 2:**
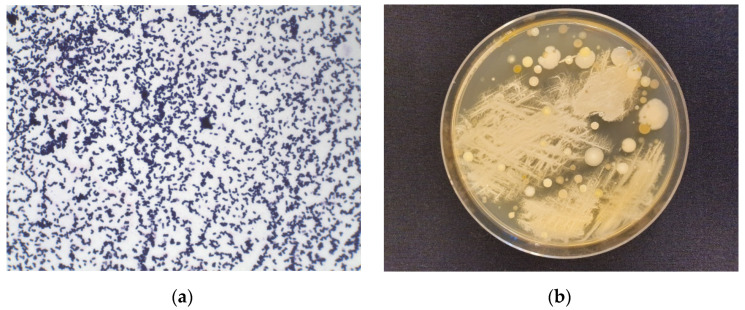
Endophytic bacteria isolated from *Marchantia polymorpha* L. (**a**) Gram straining (Gram-positive bacteria) and (**b**) Endophytes on solid Columbian agar medium.

**Figure 3:**
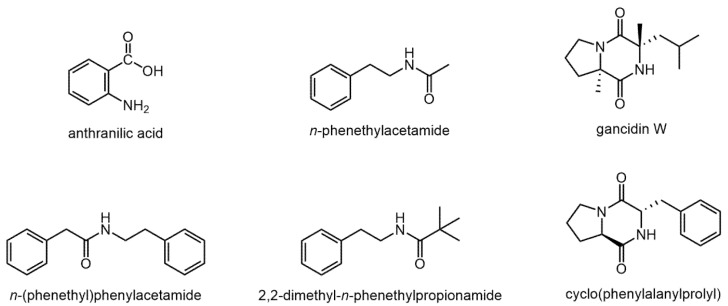
Structures of the most characteristic nitrogen containing compounds found in *M. polymorpha* endophytes.

The endophytic microorganisms were isolated from fresh thalli of *Marchantia polymorpha* L. and cultivated on Columbia agar medium (Figure 2). The volatile compounds were extracted with hexane and ethyl acetate (EtOAc). Then, chromatographic analysis of both extracts was performed. The data presented in Table 2 indicates that the ethyl acetate extract shows greater chemical diversity of metabolites compared to the hexane extract. The most characteristic components found in both extracts are nitrogen containing compounds. The structures of these compounds are presented in Figure 3. 

The major compounds identified in the ethyl acetate extract were: anthranilic acid, *N*-phenylethylacetamide, gancidine W, and cyclo(phenylalanylprolyl). None of the mentioned components were found in the hexane extract. Analysis of the hexane extract showed the presence of 2,2-dimethyl-*N*-phenylethylamide of propionic acid, also identified in the ethyl acetate extract, and *N*-(phenylethyl) phenylacetamide, which was not found in EtOAc extract. 

The presence of the same fatty acids was confirmed in both, the ethyl acetate and hexane extract, e.g., pentadecanoic and hexadecanoic acid. Significant differences were found in the case of the identified fatty acids methyl esters. Both extracts contained methyl pentadecanoate and methyl 9-hexadecanoate, while the presence of methyl hexadecanoate was noticed only in the ethyl acetate extract.

### 2.3. Cytotoxicity of Endophyte Extracts

The ethyl acetate extract obtained from *M. polymorpha* endophytes was found to possess more versatile phytochemical composition than the hexane extract and was selected for cytotoxicity studies. Furthermore, the preliminary screening showed that ethyl acetate extract exerted the most promising anticancer potential. 

The cytotoxicity of ethyl acetate extract obtained from endophyte cultures was assessed using microculture tetrazolium-based assay (MTT). The MTT test measures the activity of cellular dehydrogenases to reduce a yellow 3-(4,5-dimethylthiazol-2-yl)-2,5-diphenyltetrazolium bromide to a purple formasane crystals, which are further dissolved using mixture of sodium dodecyl sulphate, dimethylformamide and PBS. The cellular dehydrogenases retain activity only in viable, metabolically active cells, therefore, the level of enzyme activity can be used to assess the cellular viability.

**Figure 4:**
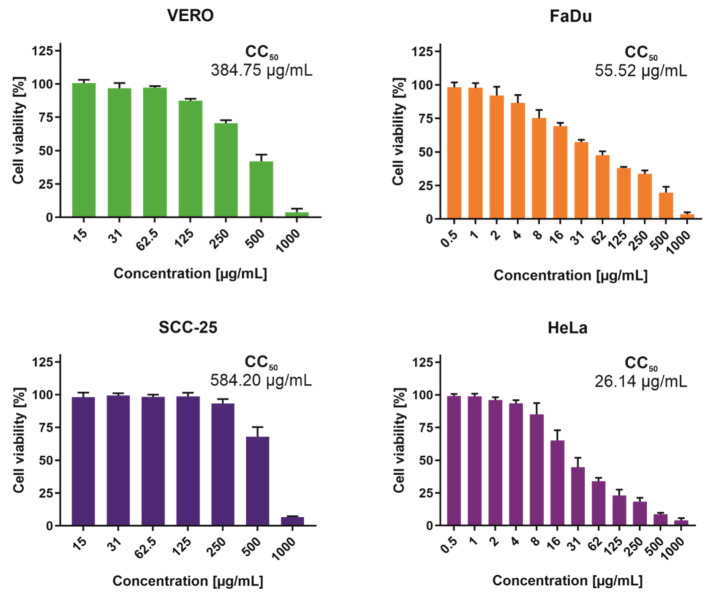
Cytotoxicity of ethyl acetate extract from *M. polymorpha* endophytes.

The panel of cell lines used in this research was comprised of normal kidney fibroblasts (VERO) and cancer cells-cervical adenocarcinoma (HeLa), and two cell lines belonging to Head and Neck Cancer Panel, namely hypopharyngeal squamous cell carcinoma (FaDu) and tongue squamous cell carcinoma (SCC-25). Selectivity Index (SI) were also calculated with reference to cytotoxicity observed for VERO cells (SI = CC_50_VERO/CC_50_CancerCells) to assess the anticancer potential of the extract. The results of cytotoxicity testing are presented on Figure 4. The highest cytotoxicity was observed on the HeLa cells with CC_50_ of 26.14 µg/mL and SI of 14.72. In the case of head and neck squamous cell lines, only FaDu showed sensitivity to ethyl acetate extract (CC_50_ 55.52 µg/mL and SI 6.93). The tongue squamous cell carcinoma derived cells were resistant to tested extract (SI 0.66).

## 3. Discussion

The vast majority of the research, conducted so far, concerns endophytic microorganisms that colonize the vascular plants. There is still a small number of studies that concern endophytes from spore-forming plants, including liverworts [12]. In case of some liverwort species, e.g., *Marchantia polymorpha*, only the diversity of endophytic microorganisms present in their tissues was determined, without the analysis of the metabolites produced by these microorganisms and their potential properties.

It has been shown that the type of endophytic microorganisms isolated from this liverwort largely depends on the subspecies and the place of sampling. The vast majority of the obtained fungal isolates belonged to Ascomycota, e.g., Sordariomycetes, Eurotiomycetes, Pezizomycetes, Saccharomycetes, Leotiomycetes, and Dothideomycetes [12]. Another study concerning *M. polymorpha* endophytes showed the presence of bacterial endophytes belonging to the genera: *Methylobacterium*, *Rhizobium*, *Paenibacillus*, *Lysobacter*, *Pirellula*, *Steroidobacter*, and *Bryobacter* [13]. However, no phytochemical studies on the endophytes of this liverwort have been conducted in comparison to *M. polymorpha* itself. This liverwort species produces many interesting compounds belonging to sesquiterpenoids and bis-bibenzyls [8,10,11]. Among bis-bibenzyls, it is worth mentioning marchantin A, the most characteristic compound occurring in this liverwort species. Marchanthin A is an aromatic compound structurally similar to tubocurarine, exhibiting myorelaxant activity. Its antioxidant and cytotoxic effects have also been confirmed [11,14,15]. Moreover, the antiprotozoal effect of marchanthin A against two strains of *Plasmodium falciparum* (NF54 and K1), as well as *Trypanosoma brucei rhodesiense*, *T. cruzi*, and *Leishmania donovani* has been demonstrated [16]. Among the volatile components present in *M. polymorpha*, cuparane-, chamigrane-, and thujopsane-type sesquiterpenoids should be mentioned [11,17,18,19,20]. GC-MS analysis of the ether extract obtained from *M. polymorpha* also confirmed the presence of compounds belonging to sesquiterpene group. The presence of cuparanes, chamigranes, and thujopsanes was confirmed. Additionally, in this Polish collection of *M. polymorpha*, acorane-type sesquitrpenoids were identified. However, metabolites with a terpene structure have not been identified in the extracts of endophyte cultures of this liverwort. Phytochemical analysis of the ethyl acetate and hexane extracts from the endophytic microorganisms of *M. polymorpha* grown on the solid Columbia agar medium was also performed using the GC-MS method. The most characteristic components found in the endophytes were nitrogen containing compounds. The presence of anthranilic acid, *N*-phenylethylacetamide, gancidine W, and cyclo(phenylalanylprolyl) has been confirmed. Cyclo(leucylprolyl) (=gancidine W), and cyclo(phenylalanylprolyl) belong to the group of bacterial diketopiperazines (DKPs). They are natural cyclic dipeptides with proven antifungal, antibacterial and antitumor properties [21]. DKPs, and especially proline derived DKPs are promising nature-inspired herbicides due to their environmental friendliness, safety, and high selectivity. Proline is linked to diverse plant stresses as defense against toxicity. It improves the formation of reactive oxygen species, signaling, and cellular apoptosis [22]. Diketopiperazines have been isolated from *Aspergillus niger*, inhabiting the liverwort *Heteroscyphus tener*. Among the isolated DKPs, heterodimers, asperazine, and asperazine A had weak impacts on human cancer cell lines PC3, A2780, K562, MBA-MD-231, and NCI-H1688 [23]. Gancidine W, in a mouse model of *Plasmodium berghei* PZZ1/100 infection, was shown to have antimalarial properties with relatively low toxicity [24]. Another study demonstrated antidiabetic activity of this compound by inhibiting α-glucosidase and α-amylase enzymes. It also exhibits free radical scavenging antioxidant activity [25].

The obtained phytochemical results cannot be compared with the literature data, because, so far, no phytochemical studies of metabolites present in endophytes of this species have been performed. There are only a few scientific publications on phytochemical studies of liverwort endophytes. These include the following species, *Scapania ciliata*, *S. verrucosa*, *Heteroscyphus tener*, *Riccardia multifida*, and *Trichocolea tomentella* [23,26,27,28,29,30,31,32,33]. As in the case of the current research, the literature data indicate no phytochemical correlation between plant material and their endophytes.

The analysis of the ether extract of *Scapania verrucosa* and the ether extract of the endophytic *Chaetomium fusiforme*, isolated from this liverwort, showed differences in the chemical composition. While the presence of sesquiterpenes (e.g., aromadendrene) is characteristic of *Scapania verrucosa*, the main metabolites produced by *Chaetomium fusiforme* are acetic acid, 3-methylvaleric acid methyl ester, and butane-2,3-diol. These components are responsible for the antifungal and antibacterial properties [26]. On the other hand, the phytochemical analysis of the endophytic fungus *Xylaria* sp., also isolated from the liverwort *Scapania verrucosa*, showed the presence of aromatic compounds including: 2,3-dimethoxy-5-methylphenol, O-methylcurvulinic acid, curvulin, methyl curvulinate, and 4-methoxycinnamic acid, among others [27]. Some endophytes inhabiting the tissues of liverworts may also be a source of new, so far unknown, me×××tabolites. From *Aspergillus sydowii*, found in *Scapania ciliata*, three new xanthone derivatives were isolated: sydoxanthone A, sydoxanthon B and 13-O-acetylsidovinin B [28].

The source of new secondary metabolites are also endophytes from the liverwort *Heteroscyphus tener*. From the culture of *Aspergillus fumigatus*, in addition to already known compounds, three new compounds were obtained, including two alkaloids, asperfumigatin and isochaetominine, and diphenyl ether, 8′-O-methylasteric acid. These compounds showed weak cytotoxic activity against the PC3 cell lines (prostate cancer) in comparison to the extract which showed a strong cytotoxicity (IC_50_ = 16.72 mg/mL) against the PC3 cell lines [29]. Bioactivity-guided fractionation of the cytotoxic extract of *Aspergillus niger*, an endophytic fungus from *Heteroscyphus tener*, afforded few naphtho-γ-pyrones, together with a cytotoxic cyclic pentapeptide, malformin A1. It shows significant in vitro cytotoxicity against the cell lines: PC3, A2780, H1688, and K562, while *A. niger* extract showed significant cytotoxicity against the HepG-2 cell line [30].

The endophytic fungus, *Penicillium concentricum*, from liverwort species *Trichocolea tomentella* showed the presence of halogenated compounds. From the ethyl acetate extract from a fermentation of the fungal strain on halogen salt supplemented rice media, which showed significant antiproliferative activity against HT-29 cells with an IC_50_ value of 2.2 μg/mL, 21 compounds were isolated [31]. It has been shown that, among all compounds isolated from *Penicilium concentricum*, the highest cytotoxic activity against various cell lines is shown by 2-bromogentisine alcohol and 3-hydroxybenzenemethanol [32].

## 4. Materials and Methods

### 4.1. Plant Material

The thalli of *Marchantia polymorpha* L. were collected in November 2020 from its natural state in the Lublin region (Cieleśnica, Biała Podlaska district, Poland) by A.L. Plant material was identified by A.L. and confirmed by Yoshinori Asakawa (Tokushima Bunri University). Voucher specimens (AL18112020-1) have been deposited in the Herbarium of Department of Pharmacognosy with the Medicinal Plant Garden, Medical University of Lublin.

### 4.2. Endophyte Cultivation

The isolation and cultivation of endophytes was carried out with the use of sterile materials under aseptic conditions. The cleaned thalli of *M. polymorpha* were immersed in 70% ethyl alcohol for 60 s. Then, after firing in the flame of a burner, it was placed in 50 mL of water and shaken with sterile metal balls to homogenize and release endophytes. The obtained supernatant was inoculated on solid nutrient media (Columbia agar). After the incubation period of 7 days, the medium was harvested with endophytes and subjected to the extraction process.

### 4.3. Extraction and Chromatographic Analysis

The fresh thalli of *M. polymorpha* were cleaned, air-dried, and homogenized in a mortar using diethyl ether (POCH S.A, Gliwice, Poland) as a solvent. The resulting supernatant was passed through a glass column filled with Celite (2 cm). The procedure was repeated until 10 mL of filtrate was obtained, which was then allowed to evaporate. The concentrated extract was subject to phytochemical analysis.

The culture of endophytes obtained from the fresh thalli of *M. polymorpha* was transferred to an Erlenmeyer flask filled with methanol (POCH S.A, Gliwice, Poland). They were then placed in an ultrasonic bath for 30 min and allowed to macerate until the next day. The procedure was repeated three times. The extract was filtered, and then concentrated under reduced pressure. A liquid–liquid extraction was performed in a 500 mL separating funnel, extracting the compounds first with hexane (POCH S.A, Gliwice, Poland) five times with 100 mL portions and then with ethyl acetate (POCH S.A, Gliwice, Poland), also with five 100 mL portions. The obtained hexane and ethyl acetate extracts were concentrated and subjected for chromatographic analysis. The GC-MS analysis of the obtained extracts was carried out with the use of a Shimadzu GC-2010 Plus gas chromatograph, coupled with a QP 2010 Ultra mass detector. Volatile compounds were separated on a ZB-5MS capillary column (Phenomenex) with a silica film thickness of 0.25 μm, column length of 30 cm, and internal diameter of 0.25 mm. The initial oven temperature was 50 °C, with a 3 min holding time, and then increased to 250 °C at 5 °C/min, and held for 15 min at 250 °C. An injection temperature of 280 °C was maintained, and helium (1 mL/min) was used as a carrier gas. The QP 2010 Ultra mass spectrometer worked in the electron ionization (EI) mode. Ionization energy was 70 eV, the scan rate was 0.2 s/scan, and the scan range was 40–500 amu. The temperature of the ion trap was 220 °C, while the injection and interface temperature was 250 °C. The injection volume was 1 µL. The sample was dosed in split mode (1:20). The retention indices (RI) of the volatile compounds present in the chromatograms were calculated with respect to the homologous series of *n*-alkanes (C6–C27). The identification of compounds was made using a computer spectral library (MassFinder 2.1, NIST 2011), mass spectra of reference compounds, as well as available literature data.

### 4.4. Cytotoxicity Testing

The VERO (ECACC, No. 84113001, green monkey kidney) were cultured using DMEM (Corning, Tewksbury, MA, USA), whereas cancer derived cell lines—FaDu (ATCC, Cat. No. HTB-43, hypopharyngeal squamous cell carcinoma) and HeLa (ECACC, Cat. No. 93021013, cervical adenocarcinoma) were grown using MEM (Corning), and in case of SCC-25 (ATCC, CRL-1628, tongue squamous cell carcinoma), using DMEM-F12. Cell media were supplemented with fetal bovine serum (FBS, Biowest) and antibiotics (Penicillin-Streptomycin Solution, Corning), and in case of SCC-25, with hydrocortisone (Sigma-Aldrich, St. Louis, MO, USA). Cell culture experiments were carried out at 37 °C in the 5% CO_2_ atmosphere (CO_2_ incubator, Panasonic Healthcare Co., Ltd., Tokyo, Japan). The DMSO (dimethyl sulfoxide) and 3-(4,5-dimethylthiazol-2-yl)-2,5-diphenyltetrazolium bromide (MTT) were purchased from Sigma, whereas phosphate buffered saline (PBS) and trypsin from Corning.

The extracts obtained from endophyte cultures were dissolved in DMSO (50 mg/mL) and filtered using a syringe filter (pore diameter 0.2 µm) to obtain stock solutions used in further in vitro studies. Stock extracts were stored frozen until used in experiments.

The cytotoxicity testing was carried out using microculture tetrazolium assay (MTT). Briefly, the cells were passaged into 96-well microculture plates to produce monolayers. After an overnight incubation, the media was removed and serial dilutions of stock extracts, ranging from 1000 to 0.98 µg/mL, were added and plates were further incubated for 72 h. The cytotoxicity of DMSO used as a solvent was also evaluated. Subsequently, media was removed, the remaining monolayers were washed with PBS and 100 µL of media supplemented with 10% of MTT solution (5 mg/mL) was added to each well and incubation continued for the next 4 h. Finally, 100 µL of SDS/DMF/PBS (14% SDS, 36% DMF, 50% PBS) solvent per well was added and after overnight incubation the absorbance at 540 and 620 nm was measured using Synergy H1 Multi-Mode Microplate Reader (BioTek Instruments, Inc. Winooski, VT, USA) equipped with Gen5 software (ver. 3.09.07; BioTek Instruments, Inc.) and data was exported to the GraphPad Prism (v7.04) for further analysis. Based on the comparison of absorbance recorded for control cells and endophyte extract treated cells, the values of CC_50_ were calculated. The CC_50_ is the concentration of tested extract decreasing the viability of appropriate cell line by 50%.

## 5. Conclusions

The results presented in this paper prove how important the source of metabolites are to endophytic microorganisms. It is also very important that there are many plant species for which endophytes and the compounds produced by these microbes have not yet been analyzed. The first phytochemical analysis of the metabolites present in the endophytes of the liverwort *Marchantia polymorpha* allowed the identification of interesting compounds. Due to the completely different type of the identified metabolites found in the endophytes of the *M. polymorpha* compared to those identified in the lichen, further research is necessary. The cytotoxicity of ethyl acetate extracts from endophytic microorganisms revealed significant anticancer potential towards hypopharyngeal squamous cell carcinoma and cervical adenocarcinoma.

## Figures and Tables

**Table 1:** The list of the compounds identified in the diethyl ether extract obtained from the thalli of *Marchantia polymorpha* L. (MaPo); RI-retention index on ZB-5 column.

Compound	RI	MaPo
7-epi-α-Cedrene	1405	tr
Italicene	1406	tr
α-Microbiotene	1421	tr
β-Funebrene	1427	tr
Thujopsene	1438	1.3
Isobazanene	1440	0.7
(*E*)-β-Farnesene	1452	0.8
Myltayl-8(12)-ene	1457	0.6
10-epi-Muurola-4,11-diene	1466	tr
β-Acoradiene	1469	0.7
β-Microbiotene	1472	0.8
Selina-4,11-diene	1475	0.9
α-Neocallitropsene	1478	3.2
β-Chamigrene	1485	4.9
α-Selinene	1499	tr
α-Cuprenene	1504	tr
β-Alaskene	1506	1.0
Cuparene	1512	11.7
β-Curcumene	1537	1.1
δ-Cuprenene	1551	2.7
Cyclopropanecuparenol	1593	10.8
*epi*-Cyclopropanecuparenol	1596	22.3
222[M]+, 111(100), 94(74), 69(73), 55(49), 43(51)	1637	2.4
220[M]+, 94(100), 111(84), 69(89), 55(41), 43(21)	1647	1.9
222[M]+,109(100), 95(96), 84(94), 55(60), 43(58)	1668	7.0
Acorenone B	1695	2.6
222[M]+, 111(100), 93(39), 83(32), 55(25), 41(15)	1702	1.6
218[M]+, 147(100), 119(68), 91(43), 55(33), 41(38)	1708	1.3
β-Herbertenol	1780	tr
Cuparophenol	1790	0.8
*ent*-9-*oxo*-α-Chamigrene	1806	0.7
Neophytadiene (Isomer 2)	1835	1.0
256[M]+, 73(100),213(31), 129(67), 57(92), 43(73)	1966	2.1
Phytol	2109	1.5
280[M]+, 79(100), 95(56), 67(66), 55(88), 41(58)	2141	0.9

tr—traces (<0.2%).

**Table 2:** The list of the compounds identified in the endophytic microorganisms of *Marchantia polymorpha* L.; RI-retention index on ZB-5 column.

Compound	RI	Hexane Extract	Ethyl Acetate Extract
Anthranilic acid	1415	−	+
*N*-Phenetylacetamide	1508	−	+
2,2-Dimethyl-*N*-phenethylpropionamide	1719	+	+
Pyrrolidino [1,2-a]piperazine-3,6-dione	1764	−	+
Cyclo(leucylprolyl) = Gancidin W	1929	−	+
*N*-(Phenethyl)phenylacetamid	2128	+	−
Cyclo(phenylalanylprolyl)	2377	−	+
Pentadecanoic acid	1828	+	+
Hexadecanoic acid	1963	+	+
Methyl pentadecanoate	1783	+	+
Methyl hexadecanoate	1884	−	+
Methyl 9-hexadecenoate	1903	+	+
Pentadecane	1497	−	+

+/−: presence/absence of a compound.

## Data Availability

The data presented in this study are available on request from the corresponding author.
